# Drug–Microbiota Interaction in Colon Cancer Therapy: Impact of Antibiotics

**DOI:** 10.3390/biomedicines9030259

**Published:** 2021-03-05

**Authors:** Ali Mohamed, Harry Menon, Marina Chulkina, Nelson S. Yee, Irina V. Pinchuk

**Affiliations:** 1 Division of Hematology-Oncology, Department of Medicine, Penn State Health Milton S. Hershey Medical Center, Penn State Cancer Institute, Pennsylvania State University College of Medicine, Hershey, PA 17033, USA; amohamed1@pennstatehealth.psu.edu (A.M.); hmenon@pennstatehealth.psu.edu (H.M.); 2 Mechanisms of Carcinogenesis Program, Division of Gastroenterology, Department of Medicine, Penn State Health Milton S. Hershey Medical Center, Penn State Cancer Institute, Pennsylvania State University College of Medicine, Hershey, PA 17033, USA; mchulkina@pennstatehealth.psu.edu; 3 Next-Generation Therapies Program, Division of Hematology-Oncology, Department of Medicine, Penn State Health Milton S. Hershey Medical Center, Penn State Cancer Institute, Pennsylvania State University College of Medicine, Hershey, PA 17033, USA

**Keywords:** antibiotics, chemotherapy, colon cancer, microbiota

## Abstract

Colon adenocarcinoma is one of the most common malignancies, and it is highly lethal. Chemotherapy plays an important role in the treatment of colon cancer at various stages of the disease. The gut microbiome has emerged as a key player in colon cancer development and progression, and it can also alter the therapeutic agent’s efficacy and toxicities. Antibiotics can directly and/or indirectly affect the balance of the gut microbiome and, therefore, the clinical outcomes. In this article, we provided an overview of the composition of the gut microbiome under homeostasis and the mechanistic links between gut microbiota and colon cancer. The relationship between the use of oral antibiotics and colon cancer, as well as the impact of the gut microbiome on the efficacy and toxicities of chemotherapy in colon cancer, are discussed. Potential interventions to modulate microbiota and improve chemotherapy outcomes are discussed. Further studies are indicated to address these key gaps in the field and provide a scientific basis for the design of novel microbiota-based approaches for prevention/use as adjuvant therapeutics for patients with colon cancer.

## 1. Introduction

Colon cancer is one of the most prevalent malignancies worldwide, and it is one of the leading causes of cancer-related death among both men and women [1]. Early-stages of colon cancer are often treated with surgical resection, with or without systemic chemotherapy in the adjuvant setting to reduce the risk of recurrence. Colon cancer remains a prevalent and difficult entity to treat in the advanced stages. Despite the evolving improvement in surgical techniques and targeted therapy, systemic chemotherapy continues to be the main treatment of choice. The backbone of systemic therapy remains 5-fluorouracil (5-FU), an inhibitor of thymidylate synthase that produces cytotoxic effects in the tumor. 5-FU is often combined with the DNA-adducting agent oxaliplatin or the topoisomerase-1-binding drug irinotecan to form the standard chemotherapy regimen(s) for metastatic colon cancer [2]. However, there have been many instances of patients who have displayed primary refractory disease in the face of frontline chemotherapy.

In recent years, several lines of evidence have suggested that the intestinal microbiome may impact the outcomes of the chemotherapy, particularly drug efficacy and toxicity [2]. Thus, further understanding of the interaction between the gut microbiome and therapeutic agents in colon cancer can help optimize these therapies. In this report, we present an up-to-date overview of the current knowledge of the microbiome in normal colon epithelia and sporadic colon cancer. The relevance of the intestinal microbiome to the outcome of standard care chemotherapy, as well as the impact of antibiotics, are discussed.

## 2. Gut Microbiome Composition under Homeostasis

The human microbiome is composed of different microorganisms, including fungi, bacteria and archaebacteria, viruses and phages. In the normal bacteriome, the total amount of bacteria in a 70 kg person is estimated to be around 3.8 × 10^13^ [3]. During the last decade, comprehensive data have been acquired on the types and ratios of microbes that inhabit the healthy human gut. Fecal microbiome analysis demonstrated that a healthy human gut bacteriome consists of 8 phyla, 18 families, 23 classes, 38 orders, 59 genera and 109 species [4]. It is dominated mostly by bacteria belonging to the Firmicutes, actinobacteria and Bacteroidetes phyla [4]. More than half of Firmicutes are members of the Clostridia (20.3%), which is the most abundantly represented class, followed by Bacteroidia (18.5%), Bifidobacteriales (16.6%), Enterobacterales (14%) and Lactobacillales (14%) [3,4]. The majority of the Clostridia class reported to be members of Clostridiales order, and all of Bacteroidia belongs to Bacteroidales; these two are the most abundant orders. Bifidobacteriales class is mostly represented by the Bifidobacteriaceae family, where strains of the *Bifidobacterium longum* are most abundant [4]. The function and interactions of these bacteria on the species and strain levels are far from complete understanding. It is clear that these bacterial classes play a prominent role in the gut homeostatic “biofilm” and regulation of gut physiology by interacting with each other as well as with the immune and non-immune host cells. These bacteria likely act as sensors and triggers for both genetic and environmental cues.

Dietary reports have shown that *Bifidobacterium* abundance is positively correlated with vegetable protein and dietary fiber intake. Moreover, *Akkermansia* has been shown to be positively associated with saturated fat intake and is negatively correlated with uptake of polyunsaturated fatty acids [4]. Overall, reciprocal communication between microbiome, host, and environment regarding changes in homeostasis results in a number of effects, both locally and in the entire system [5]. The local effects of the microbiome interplay with host and environment include nutrient absorption, synthesis of metabolites, local tolerance and inflammation, and tissue development. The systemic effects of this interplay include modulation of metabolism, systemic inflammation, immunity and tolerance [5]. Dysregulation of all these processes plays a critical role in the mechanisms of neoplastic development and progression, as well as the outcome of chemotherapy.

## 3. Mechanistic Links between Microbiota and Colon Cancer

The microbiome and its influence on carcinogenesis have been a growing area of interest and research. Accumulating evidence indicates that microbial dysbiosis of the gut contributes to cancer initiation and progression, especially in colorectal cancer [6,7]. The mechanisms by which the microbiota potentiate carcinogenesis range from the production of carcinogenic toxins to manipulation of the inflammatory and tolerogenic pathways [5,8]. Several bacterial species have shown cancer-promoting effects via dysregulation of the signaling pathways within the colonic epithelium and immune cells [7,9]. The driver-passenger model has been proposed [10], describing the existence of certain bacteria with special virulence traits that contribute to the neoplastic epithelial transformation by creating DNA damage and driving genomic instability. Examples of such bacteria are genotoxic strains of *Bacteroides fragilis* and *Escherichia coli* spp. [6,7,10].

*Escherichia coli* can produce colibactin, which causes double-strand DNA damage and potentiates intestinal tumorigenesis in mice, while *B. fragilis* toxin causes high levels of reactive oxygen species found in host cells with DNA damage [7]. Subsequently, drivers of neoplastic transformation are partially or completely replaced by opportunistic bacteria (passengers) with tumor progression-promoting properties, such as *Fusobacteria nucleatum* [9]. *F. nucleatum* can selectively interact with E-cadherin, which may amplify the development of colon cancer [7]. It has been shown that antitumor T cell-mediated adaptive immunity can be inhibited by *F. nucleatum* via expansion of the myeloid-derived suppressor cells [9,11].

Meta-analysis has confirmed that colon tumors are enriched for invasive biofilms (particularly on right-sided colon tumors) in which opportunistic bacteria with capacity for tumorigenesis (such as *B. fragilis* and/or *E. coli*) and oral pathogens, particularly *F. nucleatum,* are prevalent [12,13]. Additionally, several other taxa of oral bacteria were reported to be found in colon cancer samples differentially abundant in colon cancer compared with controls, for example, *Streptococcus* and *Prevotella* spp. A classification model of oral swab microbiota distinguished individuals with colon cancer or polyps from controls (sensitivity: 53% (colon cancer)/67% (polyps); specificity: 96%) [14]. Combination of the data from fecal microbiota and oral swab microbiota increased the sensitivity of this model to 76% (colon cancer)/88% (polyps) [14]. The mucus-invasive bacterial biofilms were identified on the colon mucosa of approximately 50% of colon cancer patients and approximately 13% of healthy subjects [13]. Using animal models of colon cancer, Drewes JL et al. [13] demonstrated that human colon cancer biofilms comprise microbial communities enhancing carcinogenesis. Interestingly, another study has shown that a high abundance of Lachnospiraceae was negatively associated with the colonization of colonic tissue with oral-like bacterial networks suggesting a protective role for certain microbiota types against colon cancer and suggested to confer resistance to colon colonization by colon cancer-promoting oral bacteria [14].

One of the intriguing areas that warrant a further investigation is how variation in tumor genetic mutations influence the cancer-associated microbiome. Current limited knowledge in this area was recently elegantly reviewed by Burns M.B. et al. [15]. Thus, herein we will just provide a brief overview. Using genome-wide techniques, several studies have shown that human genetic variants can influence microbiome composition in specific disease contexts, such as inflammatory bowel diseases and cancer [15,16,17]. Recent studies suggest that variation in tumor mutations may differentially impact the composition of the microbiota at the tumor site [12,15,18]. It has been noted that changes in the colon cancer tumor stage are associated with changes in the microbiome. For instance, while bacteria of the *Propionibacterium* genus were dominant in low-stage tumors, the bacteria of the *Granulicatella* genus prevail in late-stage tumors [18]. Interestingly, the same research team suggested that the microbiome composition profiles can predict the existence of loss-of-function mutations in the following genes APC, ANKRD36C, CTBP2, KMT2C, and ZNF717. A high abundance of the bacteria of *Bacillus* genus has been found in tumors positive for APC mutations, while the bacteria of *Ruminococcus* genus was dominant in tumor positive for KMT2C mutation. Bacteria of the r Solirubrobacterales and *Dorea* taxa were predominant in tumor-bearing ANKRD36C mutation. Bacteria of the *Filifactor* genus dominated tumor positive for CTBP2 mutation [18]. While the functional significance of these differences remains to discover, the same team has demonstrated that an increase in the above-mentioned bacterial taxa was not only associated with the genetic mutations but as well correlated with activation of the cancer-promoting pathway. In particular, an increase in bacteria of the *Bacillus* genus was also associated with the activation of the Wnt signaling pathway [18]. Additionally, it has been demonstrated in mouse models of colon cancer that Apc mutation is a prerequisite genetic defect for the *F. nucleatum* to promote tumor growth [19].

Analysis of the overall functional significance of changes in gut microbiome in colon cancer progression is complex since information on the environmental factors, including diet, medication, medical history, and other life-history traits, should be incorporated. However, over the last decade, significant progress has been made in understanding the potential signaling pathways by which individual cancer-promoting bacteria contribute to cancer progression. For example, great progress was made in understanding the role of genotoxic strains of *Bacteroides fragilis* in colon cancer initiation. This bacterium is considered to be among the driver of colon cancer and is known to form a biofilm during colonization of the colon, producing genotoxic toxins and increases the expression of cyclooxygenase (COX)-2 release of PGE_2_ [20]. *B. fragilis* toxin degrades E-cadherin, upregulates ROS production, promoting irreversible DNA damage, thus, contributing to colon cancer initiation. These mechanisms were recently nicely overviewed in-depth by Fiorentini C. et al. [21]. Some cancer-promoting strains of *Escherichia coli* sp. produce genotoxin colibactin, which is reported to contribute to genomic instability and tumor-promoting inflammation during cancerogenesis [21]. Cancer passenger bacterium *F. nucleatum* has been shown to promote colon cancer by activation of E-cadherin/β-catenin signaling via FadA adhesin [22]. It was also shown that *F. nucleatum* protected the tumor cell from immune cell attack by Fap2/TIGIT signaling, resulting in inhibition of NK cell cytotoxicity and antitumor activity of T cells [23]. Finally, a recent study by Yu M.R. et al. suggested that *F. nucleatum* well promotes epithelial to mesenchymal transition during colon cancer progression [24]. Using the cancer cell line and the AOM-DSS model of colitis-associated colon cancer, this group demonstrated that when used with epithelial barrier disruptive agent DSS, *F. nucleatum* synergistically increased the aggressiveness and EMT characteristics of cancer cells and this process involved EGFR signaling pathways [24]. Finally, the impact of the microbiome on the human epigenome was recently nicely overviewed by Dr. J. Allet and Dr. C. Sears [25]. The majority of these studies so far have focused on the role of the microbiome in the epigenetic remodeling of cancer epithelial cells. Studies in the past four years have demonstrated that gut microbes impact DNA methylation, chromatin remodeling as well non-coding RNA expression in colonic cancer epithelial cells. Among the important pathways that can be altered by the cancer-promoting microbiome are WNT signaling and overall cancer cell proliferation pathways [25]. Despite this progress in our understanding of the mechanism(s) by which microbiota contribute to cancer initiation and progression, a further in-depth study is needed to understand the role of these bacteria in the tumor-promoting microenvironment.

Finally, while studies over the last decade clearly have clearly demonstrated the presence of cancer-promoting microbiome, the detailed mechanism of the bacteria-mediated cancer-promotion remains to be elucidated; it has been suggested that these bacteria may cooperate with each other during the invasion of host tissues, evade host immune system, and maintain tumor-promoting inflammation. These events occur through a process called quorum sensing, contributing to the formation of the cancer biofilm. How to interrupt these processes is believed to be important for the next generation of anticancer drug development [8]. However, very little is known about how cancer-promoting dysbiosis occurs within colonic mucosa.

## 4. Link between Oral Antibiotic Use and Colon Cancer

The composition and complexity of the microbiome vary across individuals, and they contribute to a certain level of resilience against external perturbation. However, antibiotics are among the major agents that can perturb the normal microbiome resulting in the event known as dysbiosis [26,27].

In fact, recent studies demonstrated that changes occur over time in the composition of intestinal microbiota during antibiotic-mediated dysbiosis and recovery. Different antibiotics have been shown to have different effects on the density and diversity of the microbiota. Therefore, it has been suggested that antibiotic route of administration, mechanism of excretion, bioavailability at the luminal site of the intestinal mucosa, baseline resident microbiota will likely be among the main factors contributing to the impact of a given antibiotic on the gut microbiota in addition to its antimicrobial spectrum [26]. For example, elegant work by Zhang et al. [27] demonstrated that oral administrations of high doses of ampicillin or tetracycline expand antibiotic-resistant bacterial pool in the gut microbiome. In contrast, significantly less (ampicillin treatment) or delayed expansion of antibiotic-resistant bacteria (tetracycline treatment) was observed when these antibiotics were administrated intravenously. Interestingly to note that ampicillin is predominantly excreted by renal clearance, while tetracycline is excreted mostly biliary and renal route. While the limitation of this study was the analysis of the microbiome only in fecal samples, it strongly supports the idea that the route of administration and excretion likely to impact the colonization of the gut mucosa by antibiotic-resistant bacteria [27]. Similar observations have been made recently in poultry, where administration of ampicillin by oral gavage but not intramuscular injection resulted in overgrowth of Gram-negative β-lactam resistant microbiota [28].

It also has been shown that antibiotics may have both short- and long-term impacts on the human microbiome [29,30]. For example, while broad-spectrum antibiotic vancomycin has been shown to profoundly disrupt resident microbiota in the long-term [31,32]. Metronidazole, which shown greater intestinal absorption, metronidazole only transiently perturbed microbiome [32]. Although the exact impact of this type of exposure on colon cancer remains to be elucidated, these data suggest that even short-term treatment with antibiotics is able to shift gut microbiota to a long-term dysbiosis, which may promote the development and aggravation of several diseases. This likely to be the case of colon cancer. Thus, these data warrant use with caution administration of antibiotics that potentially could directly aggravate the overgrowth of cancer-promoting bacteriome.

**Figure 1:**
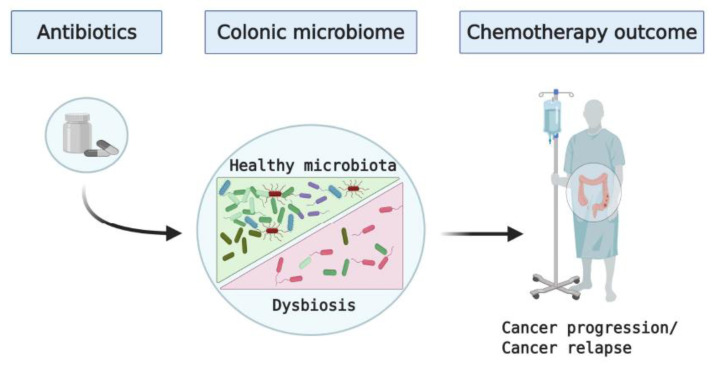
Schematic outlining potential impact of antibiotic use on the colonic microbiota and outcomes of chemotherapy in patients with colon cancer.

Additionally, the indirect impact of antibiotics on predisposition to colon cancer should be considered. While less explored area in colon cancer, it has been shown that early-life exposure to β-lactam antibiotic amoxicillin, while only transiently affecting gut microbiota, resulted in a dramatic alteration of immune responses in piglets [33]. Recently, published retrospective studies have shown a connection between oral antibiotic use, particularly the β-lactam antibiotics, and increased risk of colon cancer [34,35,36]. While the currently published data are mostly observational and they do not establish a causal relationship between oral antibiotic use and colorectal cancer, these publications support the notion that antibiotics-induced dysbiosis promotes cancer development and progression (Figure 1, Table 1). In fact, common features of post-antibiotic dysbiosis include a loss of taxonomic and functional diversity combined with reduced colonization resistance against invading pathogenic and opportunistic microorganisms [37]. All of the above is seen in colon cancer: reduction in bacterial diversity, colonization of colonic mucosa by the genotoxic strains of *E. coli* and *B. fragilis,* as well by the oral pathogens, including fusobacteria [8,12].

Another important aspect currently disregarded in the field is antibiotic resistance of the cancer-promoting bacteria. Antibiotic resistance, in particular to the β-lactam antibiotics, is surprisingly common among fusobacteria [38]. In contrast, the normal gut microbiota is often sensitive to these antibiotics [39]. One recent report observed that the frequency of the β-lactam-resistant *E. coli* is high (up to 77%) in patients with solid tumors [40]. Indeed, a positive correlation between this group of antibiotic use and colon cancer development was observed [34,35,36,37,38,39,40,41,42,43,44,45,46]. It is likely that treatment with this group of antibiotics contributes to the enrichment of colonic microbiota with pathogenic strains of *E.coli*, as has been seen in other diseases/conditions [39]. Antibiotic-induced dysbiosis has been suggested to enable *E. coli* to expand to high densities in the inflammatory models [41]. This idea is supported by recent studies showing that the majority of the *Fusobacterium* spp. are known to produce β-lactamases, the primary cause of bacterial resistance to β-lactam antibiotics [42]. Thus, it is likely that increased resistance of cancer-promoting bacteria to antibiotics provides a selective advantage for cancer-promoting bacteria to colonize colonic mucosa. This, in turn, contributes to the poor efficacy of chemotherapy used as a standard of care for patients with colon cancer. Thus, further study is needed to evaluate the mechanistic role of antibiotics in cancer-promoting dysbiosis with respect to poor chemotherapy outcomes.

## 5. Impact of the Gut Microbiome on the Efficacy and Toxicities of Chemotherapy in Colon Cancer

Over the last decade, published reports indicate that the composition of the gut microbiome impacts the host response to the outcome of chemotherapy. *F. nucleatum* has been shown to promote resistance to 5-FU and oxaliplatin via activation of autophagy [43] and an increase in the expression of antiapoptotic protein BRC3 [44] in colon cancer cells through TLR4 dependent signaling. A recent study by Yuan L. et al. also has shown that β-lactam/streptomycin-induced dysbiosis resulted in overgrowth of enterobacteria and reduced efficacy of the 5-FU therapy in a preclinical model of colon cancer [45].

Gut microbiota-mediated metabolism is known to promote pharmacologic effects and enhance absorption of several orally administered drugs, including chemotherapeutics [46]. The rate and extent of the impact of the gut bacterial metabolism on the activity, bioavailability, and toxicity of oral drugs depend on the amount of drug that reaches the distal part of the gastrointestinal tract. Some drugs have little contact with the small intestinal/colonic microbiota because they are rapidly and completely absorbed in the upper gastrointestinal tract. Some other drugs are transformed to active, inactive, or toxic metabolite(s) by the microbiota [46,50,51,52]. Chemotherapy-induced diarrhea (CID) and mucositis are among the most common dose-limiting side effects. For example, regimens of fluoropyrimidines (5-fluorouracil, capecitabine) and irinotecan are associated with about 80% CID [52,53,54].

While the mechanisms of CID are not fully delineated, it is currently known that mucositis and CID induced by irinotecan involve the metabolic activity of the gut microbiota. Irinotecan is hydrolyzed in the liver to form the active metabolite SN-38, which exhibits antitumor activity. As part of the detoxification process in the liver, SN-38 is metabolized by UDP glucuronosyl-transferase 1A1 to form the inactive SN-38G. It is then excreted to the intestine via the bile duct and deconjugated to SN-38 by the β-glucuronidases produced by intestinal bacteria causing CID [46,52]. For instance, with the administration of irinotecan, Proteobacteria tend to increase in the gut flora [55]. Proteobacteria expresses β-glucuronidase, which may be able to increase the active metabolite of irinotecan, SN-38, by cleaving the glucuronide moiety for use as a carbon source [55]. Despite these advances in the field, further mechanistic studies are necessary to understand the interplay between gut microbiota and chemotherapeutic drugs. The goal is to improve the efficacy and reduce the toxicity of the current standard-of-care chemotherapy for patients with colon cancer.

## 6. Potential Interventions to Modulate Microbiota and Improve Chemotherapy Outcomes

**Figure 2:**
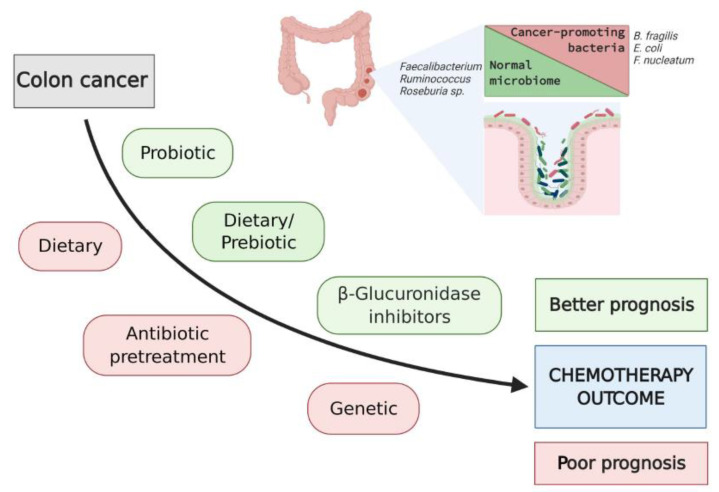
A schematic to illustrate approaches to modify intestinal microbiota and improve clinical outcomes of chemotherapy in patients with colon cancer.

While it may not be feasible to modify the genetic influence in developing colon cancer in conditions, such as mutation of the *APC* gene or the mismatch repair genes, as seen in familial adenomatous polyposis (FAP) syndrome and Lynch syndrome, respectively, these genetic syndromes are relatively rare [56]. Contrary to the FAP and Lynch syndrome, the majority of colon cancer cases are sporadic, where the environmental factors and their impact on microbiota if clearly identified and are likely to be adjustable. Potential environmental modulators of the gut microbiome composition include antibiotics, diet, use of pre-/probiotics, exercise and sleep cycles [57]. A schematic overview of potential approaches to modify intestinal microbiota and improve outcomes of chemotherapy in patients with colon cancer is presented in Figure 2.

The use of antibiotics, such as neomycin to treat irinotecan-induced diarrhea in patients with cancer has shown some promise [46]. However, a study in preclinical models revealed that antibiotic administration, including neomycin in the context of colon cancer chemotherapy, such as oxaliplatin, has led to the decreased antitumor activity of oxaliplatin [58]. These results indicate that there is a substantial mechanism within the gut microbiome that modulates chemotherapy efficacy. Thus, taken together with the role of antibiotics-induced dysbiosis in the development of colon cancer, antibiotics use in cancer patients should be taken with caution. Other alternatives to improve/prevent chemotherapy-induced toxicity have been proposed over the last few years: (1) use of pro- and prebiotic shown promise in a preclinical model and clinical trial [8,45,59]; (2) use of small molecule inhibitors that targeted bacterial β-glucuronidase in vitro and in preclinical models [52]. Remarkably, the use of the inhibitors of bacterial β-glucuronidase in vivo has been shown to dramatically reduce irinotecan-induced dysbiotic bloom of bacteria of the Enterobacteriaceae family [52]. The bacterial enzyme has a unique loop not found in the human form of the enzyme, which creates an opportunity for the specific inhibition of this bacterial enzyme. However, β-glucuronidases from *E. coli*, as well as other organisms such as *Streptococcus agalactiae, Bacteroides fragilis,* and *Clostridium perfringens*, have been shown to have significant differences in terms of their inhibition by small molecule inhibitors. This represents another example of the diversity in the gut microbiome [35,36,37,38].

Changes in diet have been associated with modification of the gut microbiome. Dietary consumption of food carcinogens, high-fat and red meat is known to promote colon cancer directly and indirectly through favoring outgrowth of cancer-promoting bacteria [57,60]. High-fat diet results in the enrichment of primary bile acid, which can be converted to secondary bile acid via complex microbial biotransformation. The increased secondary bile acids alter the gut microbiota composition, leading to cancer-promoting microbial dysbiosis, an increase in inflammatory damage, and tumor formation [61,62]. In fact, fecal levels of secondary bile acids correlate with mucosal and metabolic markers of the risk of developing colorectal cancer in adults. However, this risk can be modified within a few weeks by implementing dietary change [61,63].

Population-based studies suggest that individuals consuming the highest intakes of dietary fiber have reduced risks of colorectal adenoma and distal colon cancer as well as improved chemotherapy outcomes [64,65,66,67]. These effects likely due to the increased production of short-chain fatty acids (SCFA), including propionate, acetate and butyrate, during microbial fermentation of the dietary fiber in the colon. SCFA has been shown to inhibit inflammatory responses through epigenetic modifications [68,69]. In particular, the SCFAs, including valerate (C5), butyrate (C4), and to a lesser extent propionate (C3) and acetate (C2), downregulate inflammatory responses through inhibition of histone deacetylation and increase in histone acetylation. This likely contributes to the prevention or reduction of chromatin remodeling in tumor cells and their microenvironment [70,71]. Despite advances in the understanding of the beneficial effect of SCFA producers on the gut mucosa, it is not well understood how these bacteria and their metabolites affect tumor-promoting inflammatory responses within the tumor microenvironment.

The normal SCFA-producing microbiota belongs mostly to the *Ruminococcus*, *Roseburia*, *Faecalibacterium*, and *Bifidobacterium* genera [5,6,7,60]. A recent study using the preclinical model of colitis-associated colon cancer (AOM-DSS model of colon cancer) demonstrated the preventive and therapeutic potential of the SCFAs [72]. Despite these promising results, current data remain somewhat contradictory. There are also some limitations in the methodology used for tissue/fecal sample preparation and analysis of the SCFAs, particularly the use of frozen samples and the high-performance liquid chromatography (HPLC) method [73] for the detection of SCFAs. Another recent study [74] focused on insulin resistance has shown that circulating instead of fecal SCFAs are directly linked to metabolic health. This suggests that measuring circulating SCFA may have the potential as a biomarker/mediator of SCFA producing bacteria effects on the host. On the other hand, lower anticancer metabolites were noted in patients on a high-protein–low-carbohydrate diet [75]. High-fat and high-protein diets cause reductions in SCFA metabolism, and SCFA-producing microbiota has been shown to be reduced by irinotecan in a preclinical study [48]. Further mechanistic studies are in need to determine the therapeutic potential of manipulating the high fiber diet-microbiota-SCFA interplay in colon cancer and its chemotherapy.

The use of prebiotic is another attractive approach for cancer prevention and improvement of chemotherapy outcomes. Prebiotics is classically defined as nondigestible food ingredients that provide substrates selectively utilized by host microorganisms to confer health benefits [60]. The use of prebiotics oligofructose and inulin in conjunction with cytotoxic drugs, including 5-FU, has been shown to increase the lifespan of the experimental animals in preclinical models of metastatic solid tumors [76,77]. Preclinical and clinical studies have shown that the use of indigestible fiber-based prebiotic increased the abundance of the normal microbiota of *Faecalibacterium, Ruminococcus* and *Roseburia* spp. and their production of anticancer metabolites, such as SCFAs [60,78]. It also has been suggested that prebiotics interacts with the bacterial receptor on pathogens, preventing their adhesion to the colonic mucosa [79,80].

The use of health beneficial bacteria (a.k.a., probiotics) has been suggested to exhibit anticancer effect through different mechanisms, such as detoxification, reduction of tumor-promoting inflammation, antagonism with cancer-promoting bacteria, and secreting anticancer metabolites [14,81]. Probiotics have also been shown to have a role in decreasing mucositis. One study found that a probiotic mixture containing *Streptococcus thermophiles, Bifidobacterium breve, B. longum, B. infantis, Lb. paracasei, Lb. delbreuckii, Lb. acidophilus, and Lb. plantarum* reduced diarrhea and weight loss in rats treated with irinotecan [59]. Furthermore, in a study of 150 patients receiving 5-FU-based chemotherapy for CRC, supplementation with *L. rhamnosus* reduced the frequency of CID and abdominal discomfort, compared with guar gum fiber [82]. However, the data on the improvement of the efficacy of colon cancer chemotherapy by probiotic remain limited and contradictory. One of the *Bifidobacterium* and *Lactobacillus*-based probiotic therapy was not found to increase the efficacy of 5-FU treatment in a murine preclinical model of colon cancer, despite improving body weight and reducing dysbiosis [45].

Therefore, in-depth studies are needed using relevant animal models of colon cancer to determine the impact of pre-/probiotic use on the clinical outcomes of chemotherapy. Translation of the preclinical data to the clinic will be a challenge due to limitations of the current animal models that mimic sporadic colon cancer. The difference in the murine versus human microbiome should be taken into consideration. Additionally, the benefits of pre-/probiotic interventions in the context of colon cancer chemotherapy depends on the specific compound and strain, respectively, and it may vary among different individuals. Thus, the individual microbiome should be taken into consideration in a personalized approach when developing probiotics as supplementation of chemotherapy for colon cancer patients.

## 7. Conclusions

The role of the intestinal microbiome in malignancy continues to be elucidated through studies of the microbiome effects on the efficacy and toxicities of chemotherapy. However, despite the exponential growth in the marketing of cancer-protective microbiome, fundamental knowledge gaps exist regarding their health benefits for cancer patients, their mechanisms of action, long-term effects, and potential interactions with the host physiology. Consecutively, it remains uncertain which strains of bacteria are most appropriate for use in particular types of colon cancer. Understanding the relationship of antibiotics use and outcome of chemotherapy in colon cancer through investigation of the baseline normal/tumor microbiota is needed to improve and update guidance for antibiotics use in relation to chemotherapy in colon cancer patients. Therefore, further studies are in need to address these key gaps in the field and provide a scientific basis for the design of novel microbiota-based adjuvant therapeutics approaches for sporadic colon cancer.

## Figures and Tables

**Table 1:** Summary of studies on the role of antibiotics in colon and rectal cancers.

Type of Antibiotics	Study Type	Major Finding	Reference
Penicillin	Human, retrospective	Exposure to multiple courses of penicillin increases colon cancer risk	[34]
Overall antibiotics	Human, retrospective	Exposure to antibiotics increases the relative risk for primary colon cancer	[47]
Oral use of ampicillin/amoxicillin	Human, retrospective	Oral use of ampicillin/amoxicillin increased the risk of colon cancer	[35]
Oral use of tetracyclines	Human, retrospective	Oral use of tetracyclines reduced the risk of rectal cancer	[35]
Oral administration of the mix of antibiotics: ampicillin/neomycin/ metronidazole/ vancomycin	Animal study, azoxymethane (AOM)/dextran sodium sulfate (DSS)-induced model of colitis-associated cancer (CAC)	Pretreatment of animals with the mix of antibiotics 3 weeks prior, but not during AOM/DSS treatment, did not decrease tumorigenesis	[48]
Oral or intravenous medication of one of the seven antibiotic classes, including penicillins, cephalosporins, macrolides, tetracyclines, sulfonamides, quinolones, and nitroimidazoles	Human, retrospective	Antibiotic exposure could be during therapy with bevacizumab inversely associated with the mortality in metastatic colorectal cancer	[49]

## Data Availability

Data sharing not applicable.
